# Intensity-Specific Physical Activity and Subjective Well-Being in Older Adults With and Without Frailty

**DOI:** 10.1093/geroni/igaf122.2743

**Published:** 2025-12-31

**Authors:** Satoshi Seino, Yuri Yokoyama, Misao Kojima, Hiroki Mori, Atsuko Tanide, Takuya Ueda, Yoshinori Fujiwara, Erika Kobayashi

**Affiliations:** Yamagata University, Yamagata, Yamagata, Japan; Tokyo Metropolitan Institute for Geriatrics and Gerontology, Itabashi City, Tokyo, Japan; Tokyo Metropolitan Institute for Geriatrics and Gerontology, Itabashi City, Tokyo, Japan; Tokyo Metropolitan Institute for Geriatrics and Gerontology, Itabashi City, Tokyo, Japan; Tokyo Metropolitan Institute for Geriatrics and Gerontology, Itabashi City, Tokyo, Japan; Tokyo Metropolitan Institute for Geriatrics and Gerontology, Itabashi City, Tokyo, Japan; Tokyo Metropolitan Institute for Geriatrics and Gerontology, Itabashi City, Tokyo, Japan; Tokyo Metropolitan Institute for Geriatrics and Gerontology, Itabashi City, Tokyo, Japan

## Abstract

The health effects of physical activity (PA) may depend on both intensity and health status. This cross-sectional study examined the association between intensity-specific PA and subjective well-being (SWB) in older adults with and without frailty. This study included 32,392 non-disabled adults aged ≥65 years living in Tokyo, Japan. SWB was assessed using the Cantrill Ladder (0–10 scale). Daily durations of walking/standing (2.0 metabolic equivalents: < 1, 1–2.9, or ≥ 3 h/day) and vigorous activity (4.5 metabolic equivalents: none, < 1, or ≥ 1 h/day) were measured using the validated PA questionnaire. Frailty status was determined using the Kihon Checklist. Multivariable-adjusted partial regression coefficients for SWB were estimated for each PA intensity. Among non-frail older adults, both walking/standing and vigorous activity exhibited a positive dose-response relationship with SWB (P < 0.001 for trend), with coefficients of 0.07 (0.01–0.14) for 1–2.9 h/day and 0.18 (0.11–0.25) for ≥3 h/day of walking/standing (reference: < 1 h/day), and 0.16 (0.10–0.21) for < 1 h/day and 0.17 (0.11–0.23) for ≥1 h/day of vigorous activity (reference: no activity). Among frail older adults, walking/standing remained positively associated with SWB, with coefficients of 0.16 (0.07–0.25) for 1–2.9 h/day and 0.22 (0.11–0.33) for ≥3 h/day (P < 0.001 for trend). In contrast, vigorous activity showed a positive association only for < 1 h/day (0.13 [0.02–0.25]), with no significant association for ≥1 h/day (-0.09 [-0.22, 0.05]). While walking/standing was beneficial for SWB regardless of frailty, a frailty-dependent paradox emerged for vigorous activity. Optimizing SWB in frail older adults may require moderating vigorous activity within an appropriate range.

